# MitoQ as a Mitochondria-Targeted Antioxidant in Sperm Cryopreservation: An Updated Review on Its Mechanisms, Efficacy, and Future Perspectives

**DOI:** 10.3390/antiox14111350

**Published:** 2025-11-11

**Authors:** Abbas Farshad, Axel Wehrend

**Affiliations:** Veterinary Clinic for Reproductive Medicine and Neonatology, Justus-Liebig-University of Giessen, Frankfurter Str. 106, 34392 Giessen, Germany; axel.wehrend@vetmed.uni-giessen.de

**Keywords:** MitoQ, ROS, spermatozoa, cryopreservation, animals

## Abstract

Sperm cryopreservation is a key technique in assisted reproductive technologies (ART), livestock breeding, fertility preservation, and wildlife conservation. However, the freeze–thaw process induces significant oxidative stress through the production of reactive oxygen species (ROS) by mitochondria, which can lead to impaired sperm motility, membrane damage, DNA fragmentation, and reduced fertilization potential. MitoQ is a mitochondria-targeted antioxidant consisting of a ubiquinone moiety conjugated to triphenylphosphonium (TPP^+^). MitoQ selectively accumulates in the mitochondrial matrix, where it efficiently scavenges reactive oxygen species (ROS) at their point of origin. This targeted action helps preserve mitochondrial function, sustain ATP production, and inhibit apoptotic signaling. Extensive experimental evidence across diverse species, including bulls, rams, boars, humans, dogs, and goats, shows that MitoQ supplementation during cryopreservation enhances post-thaw sperm viability, motility, membrane integrity, and DNA stability. Optimal dosing between 50 and 150 nM achieves these benefits without cytotoxicity, although higher doses may paradoxically increase oxidative damage. Compared to conventional antioxidants, MitoQ offers superior mitochondrial protection and enhanced preservation of sperm bioenergetics. Future directions involve exploring synergistic combinations with other cryoprotectants, advanced delivery systems such as nanoparticles and hydrogels, and detailed mechanistic studies on long-term effects. Overall, MitoQ represents a promising adjunct for improving sperm cryopreservation outcomes across clinical, agricultural, and conservation settings.

## 1. Introduction

Sperm cryopreservation is a cornerstone technique in reproductive biology, providing a reliable means of long-term storage of male gametes. Since its inception, this technology has been widely adopted in assisted reproductive technologies (ART) for humans, artificial insemination in livestock, fertility preservation for cancer patients and individuals undergoing gonadotoxic therapies, and in the conservation of endangered species [1,2,3,4,5]. The fundamental principle of cryopreservation involves either controlled-rate or fast cooling of spermatozoa to ultra-low temperatures (typically −196 °C using liquid nitrogen), with “rapid” cooling referring to conventional freezing rather than vitrification, in order to halt cellular metabolism and biochemical activity. This quiescent state preserves the viability, genetic integrity, and epigenetic landscape of spermatozoa, enabling successful fertilization even years after initial collection. However, despite its versatility, cryopreservation is associated with various drawbacks. Post-thaw sperm commonly shows marked declines in motility, viability, acrosome integrity, and fertilization capacity [6,7,8]. These functional impairments are largely attributed to the biophysical and biochemical stressors imposed during the freeze–thaw cycle, including cold shock, osmotic imbalance, intracellular ice formation, and lipid phase transitions, all of which cause membrane and cytoskeletal damage [9,10]. One of the most critical consequences of cryogenic stress during sperm cryopreservation is the induction of oxidative stress, which arises primarily from an overproduction of reactive oxygen species (ROS). This imbalance can compromise membrane integrity, DNA stability, and overall sperm function, underscoring the importance of antioxidant strategies in cryopreservation protocols [11,12].

Spermatozoa are particularly vulnerable to oxidative stress because their plasma membranes are rich in polyunsaturated fatty acids (PUFAs) and they possess only limited cytoplasmic antioxidant defenses [13]. Moreover, mammalian sperm cells lack additional protective mechanisms, as they do not retain cytoplasmic organelles such as peroxisomes, key sites for detoxifying ROS through enzymes like catalase, since these structures are eliminated during spermiogenesis [14]. As a result, sperm are particularly vulnerable to oxidative damage, which can compromise their fertilization capacity and adversely affect early embryonic development. Reactive oxygen species (ROS)—including superoxide (O_2_^−^), hydrogen peroxide (H_2_O_2_), and hydroxyl radicals (•OH)—trigger lipid peroxidation, protein oxidation, DNA fragmentation, and apoptotic signaling, collectively impairing sperm function and diminishing reproductive outcomes [15,16,17]. Notably, mitochondria play a central role in this process as they act both as major sources and as primary targets of ROS. In spermatozoa, mitochondria are densely localized in midpiece, where they drive ATP production through oxidative phosphorylation, thereby sustaining flagellar motility and hyperactivation [18]. Cryogenic stress during sperm cryopreservation can disrupt mitochondrial membrane potential (Δψm), causing electron leakage from the electron transport chain (ETC) and increasing the production of ROS [19,20,21]. This mitochondrial dysfunction may activate intrinsic apoptotic pathways, contributing to further cellular damage. Therefore, maintaining mitochondrial integrity is essential for preserving sperm viability and function throughout the cryopreservation process, as demonstrated by studies highlighting the link between mitochondrial destabilization and oxidative stress in cryopreserved reproductive cells [20], and the role of ROS-induced damage as a major contributor to sperm cryoinjury and impaired motility [21].

To counter oxidative stress, researchers have explored the use of antioxidants such as vitamin E, glutathione, melatonin, and resveratrol. While these compounds show some efficacy in reducing cryodamage, their clinical utility is limited by poor mitochondrial targeting, short half-lives, and non-specific activity [22]. Consequently, attention has shifted toward mitochondria-targeted antioxidants, notably MitoQ (mitoquinone mesylate), a coenzyme Q10 derivative that selectively accumulates in the mitochondrial matrix via a lipophilic triphenylphosphonium (TPP^+^) cation, enabling efficient scavenging of ROS at their primary source [22,23,24]. Supplementation with MitoQ during cryopreservation showed a mild positive effect on sperm motility and kinetics, particularly at 25 nM, whereas a higher concentration (200 nM) negatively affected motility and viability, without altering membrane integrity, acrosome status, DNA integrity, or mitochondrial activity [25].

**Figure 1:**
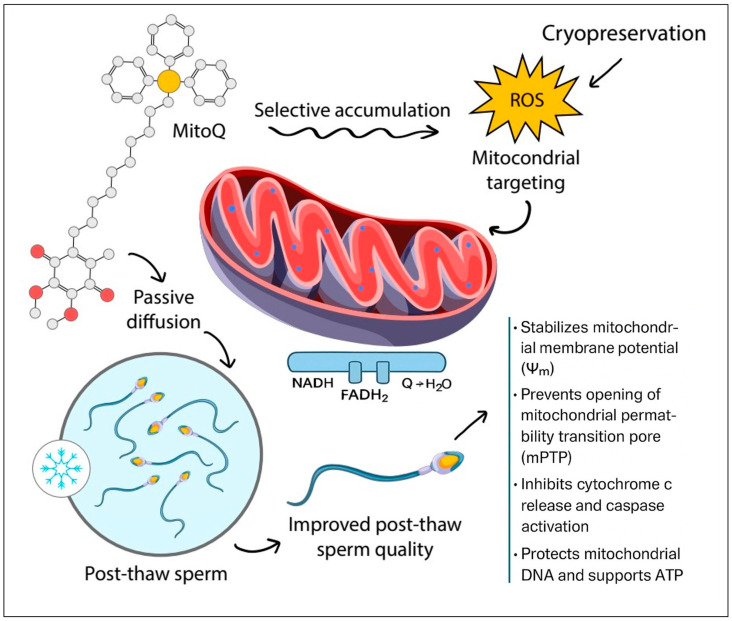
Schematic illustration created in Adobe Illustrator showing how MitoQ protects sperm mitochondria during cryopreservation. MitoQ, a mitochondria-targeted antioxidant, passively diffuses into sperm cells and selectively accumulates in the mitochondrial matrix. During cryopreservation, elevated reactive oxygen species (ROS) cause oxidative stress and mitochondrial dysfunction. By targeting mitochondria, MitoQ stabilizes the mitochondrial membrane potential (Ψm), prevents opening of the mitochondrial permeability transition pore (mPTP), inhibits cytochrome c release and caspase activation, and protects mitochondrial DNA while supporting ATP production. These actions collectively reduce oxidative damage and improve post-thaw sperm quality, motility, and viability.

Conventional coenzyme Q supplementation has shown limited success because its lipophilic nature restricts bioavailability. In contrast, mitoquinone (MitoQ) has emerged as an orally available, mitochondria-targeted variant of coenzyme Q. As a conjugate of coenzyme Q, MitoQ effectively embeds its quinone moiety within the hydrophobic core of the polarized inner mitochondrial membrane, enabling it to act as an efficient superoxide scavenger and reduce lipid peroxidation. Furthermore, endurance training has been shown to elevate coenzyme Q10 levels, which in turn supports mitochondrial function. These observations have fueled growing interest in the potential of MitoQ supplementation to enhance exercise performance [16,17,25,26,27,28]. In parallel, recent studies demonstrate that MitoQ improves post-thaw sperm function across multiple species by preserving mitochondrial membrane potential (Δψm), reducing lipid peroxidation, enhancing motility and viability, and lowering apoptotic markers [29,30,31,32], as shown in Figure 1.

As summarized in Table 1, experimental and clinical applications of MitoQ in sperm biology reveal species-specific and dose-dependent effects. For instance, in goats, nanomolar concentrations (100–150 nM) significantly improved post-thaw viability, plasma membrane integrity, and mitochondrial activity [33,34]. Similarly, comparable benefits were observed in canine sperm with antifreeze protein supplementation [35], as well as in poultry and rams, where optimal nanomolar concentrations enhanced motility, ATP levels, and preserved sperm quality during storage [36,37]. In contrast, in bulls, MitoQ failed to improve cryosurvival and even increased ROS at higher concentrations [38], highlighting the importance of precise dose calibration. In humans, increased motility but no significant effect on viability was reported [30], whereas in boar sperm, reduced lipid peroxidation and improved post-thaw viability were observed [39]. Collectively, these findings highlight both the promise and the limitations of MitoQ in sperm cryobiology. This study aims to critically evaluate the mechanistic foundations and experimental evidence surrounding MitoQ supplementation in sperm cryopreservation, with a particular focus on mitochondrial protection and functional recovery. As cryopreservation protocols increasingly adopt mechanistically guided approaches, MitoQ stands out as a potential strategy to improve reproductive outcomes in human medicine, livestock breeding, and biodiversity conservation.

## 2. Mechanism of Action of MitoQ

MitoQ (mitoquinone mesylate) is a mitochondria-targeted antioxidant designed to neutralize oxidative stress directly at its origin, the mitochondrial respiratory chain. Unlike traditional antioxidants (e.g., vitamin C, vitamin E, melatonin, glutathione), which lack subcellular specificity and exhibit poor bioavailability in spermatozoa, MitoQ penetrates mitochondrial membranes and accumulates selectively in the matrix [41]. Structurally, MitoQ consists of a ubiquinone moiety, similar to coenzyme Q10, covalently linked to a lipophilic triphenylphosphonium (TPP^+^) cation via a ten-carbon alkyl chain [23]. The TPP^+^ allows the molecule to pass through lipid bilayers and accumulate in the negatively charged mitochondrial matrix, driven by Δψm (−150 to −180 mV), reaching concentrations several hundred-fold higher than in the cytosol. Once inside the mitochondria, MitoQ is reduced by complex II (succinate dehydrogenase) of the electron transport chain to its active antioxidant form, ubiquinol [16]. Ubiquinol functions as a potent scavenger of ROS, including superoxide (O_2_^−^), hydrogen peroxide (H_2_O_2_), and peroxynitrite (ONOO^−^), all of which contribute significantly to cryodamage in spermatozoa. Importantly, MitoQ does not interfere with oxidative phosphorylation or ATP production, thereby supporting the preservation of sperm motility and viability [17].

MitoQ exerts a multifaceted protective influence on mitochondria, making it especially valuable in sperm cryopreservation protocols where mitochondrial integrity is critical for post-thaw functionality. One of its primary actions is the preservation of mitochondrial membrane potential (Δψm), which is indispensable for ATP production, calcium regulation, and ion homeostasis. Stabilization of Δψm helps sustain mitochondrial bioenergetics even under cryogenic stress [16,17]. Another crucial mechanism involves the inhibition of mitochondrial permeability transition pore (mPTP) opening. The activation of this pore is a well-established trigger for cytochrome c release and the subsequent induction of apoptosis. By preventing mPTP opening, MitoQ preserves mitochondrial integrity and enhances cell survival during and after freezing [18]. Additionally, MitoQ localizes within the mitochondrial inner membrane, where it directly scavenges ROS and prevents lipid peroxidation of PUFAs. This protection is vital for maintaining membrane fluidity, mitochondrial function, and sperm motility [19]. Unlike conventional antioxidants that require high doses and risk disrupting physiological redox signaling, MitoQ targets mitochondria directly, restoring redox balance while preserving essential signaling pathways. By neutralizing oxidative stress at its source, protecting mitochondrial integrity, and limiting apoptosis, MitoQ reduces cryo-induced damage—ultimately improving post-thaw sperm quality and fertilization potential [15].

Beyond its promising role in sperm cryopreservation, MitoQ has also been investigated in other reproductive and gamete-related settings (Table 2). Recent studies demonstrate its capacity to improve oocyte quality, fertilization outcomes, and early embryonic development by targeting mitochondrial dysfunction and oxidative stress. For instance, bovine oocyte in vitro maturation (IVM) supplemented with MitoQ resulted in enhanced maturation and blastocyst rates, along with increased mitochondrial activity and reduced ROS accumulation [42]. Similarly, MitoQ supplementation during mouse oocyte IVM under oxidative stress conditions improved spindle integrity, chromosomal stability, and overall oocyte survival [43]. Notably, the addition of MitoQ to bovine in vitro fertilization (IVF) media has been shown to enhance embryo development, improve mitochondrial function, and mitigate oxidative damage [44]. Collectively, these findings highlight the broad applicability of MitoQ across reproductive systems. By stabilizing mitochondrial function and limiting oxidative stress, MitoQ not only preserves gamete quality but also enhances downstream embryonic competence. Such results reinforce its potential as a targeted antioxidant strategy in both assisted reproduction and animal breeding technologies.

Preclinical studies in broader disease models (Table 3) provide strong support for the mechanistic actions of MitoQ. In ischemia–reperfusion injuries, MitoQ markedly reduced ROS, tissue damage, and apoptosis in rodent models affecting the liver, gut, and kidneys [45]. These protective effects are linked to MitoQ’s ability to accumulate within mitochondria, neutralize excessive ROS at the source, and preserve mitochondrial membrane integrity during acute oxidative stress. In cardiovascular models, oral administration of MitoQ improved endothelial function and attenuated cardiac hypertrophy [46]. Mechanistically, these benefits arise from restoring nitric oxide bioavailability, preventing mitochondrial-driven vascular dysfunction, and reducing oxidative damage to cardiac tissues. In reproductive biology, supplementation of human sperm with MitoQ enhanced motility and mitochondrial activity without compromising viability [42]. This indicates that MitoQ supports ATP production required for sperm motility while protecting mitochondrial DNA and proteins from oxidative damage, making it valuable for fertility preservation and assisted reproductive technologies. Beyond these systems, MitoQ has also demonstrated neuroprotective effects in models of neurodegeneration, where it reduced oxidative stress and improved neuronal resilience [47]. These outcomes likely stem from stabilization of mitochondrial dynamics, prevention of oxidative damage to neuronal membranes and support of synaptic function, which are critical in age-related cognitive decline and other neurodegenerative disorders. Taken together, these mechanistic insights highlight how MitoQ’s mitochondria-targeted antioxidant properties translate into protection across cardiovascular, reproductive, neural, and acute injury models, underscoring its broad therapeutic promise and the importance of further clinical investigation.

The broad spectrum of protective effects conferred by MitoQ holds significant promise across multiple domains of reproductive and biological science. In Assisted Reproductive Technologies (ART), MitoQ supplementation could offer transformative benefits, particularly for individuals facing infertility associated with oxidative stress or undergoing repeated IVF cycles, where sperm quality is often compromised [8,9,10,13]. By enhancing mitochondrial function, MitoQ has the potential to improve fertilization rates, embryo development, and overall pregnancy outcomes [5,12,48]. This may, in turn, reduce the need for invasive procedures or reliance on donor sperm, providing a more natural and effective route to conception [5]. In livestock breeding, MitoQ’s ability to improve post-thaw sperm motility and viability represents a valuable approach to increasing the success of artificial insemination. Its demonstrated efficacy across different species highlights its broad applicability [5,31,48]. By enhancing reproductive efficiency, MitoQ can lower breeding costs and accelerate genetic gain, thereby supporting more sustainable and productive agricultural systems. The implications extend further into conservation biology, where cryopreservation plays a vital role in safeguarding endangered species [5,34]. MitoQ’s ability to maintain sperm integrity through repeated freeze–thaw cycles support its application in wildlife conservation and ex situ breeding programs [5,34,48]. In scenarios where gamete availability is limited, MitoQ provides a critical strategy for preserving genetic diversity and advancing species restoration efforts [34].

Looking ahead, incorporating MitoQ into advanced cryopreservation strategies may further enhance its protective efficacy. Studies have shown that combining MitoQ with classical antioxidants such as vitamin E, membrane stabilizers like cholesterol-loaded cyclodextrins, or osmo-protectants such as trehalose can produce additive or synergistic effects by targeting multiple pathways of cryodamage simultaneously [20,34,35,36]. In parallel, foundational work has highlighted the potential of novel delivery systems—such as nanoparticle encapsulation, hydrogel embedding, and three-dimensional cryo-scaffolds—to enhance MitoQ’s mitochondrial targeting, chemical stability, and controlled release during both freezing and thawing phases [15,16,17,23,24]. Collectively, these innovations position MitoQ as a promising tool for advancing reproductive technologies in human medicine, livestock breeding, and biodiversity conservation [5,20,31,36,45].

## 3. Dosage and Toxicity Considerations

Although MitoQ offers compelling antioxidant benefits, its biological effects are highly dose-dependent and require careful calibration to avoid cytotoxic consequences. Research has demonstrated that concentrations ranging from 50 to 150 nM are optimal for maximizing antioxidant activity without inducing toxicity in sperm cells across various species [19]. Within this effective range, MitoQ efficiently scavenges mitochondrial ROS, thereby preserving essential sperm functions such as motility and membrane integrity. However, exceeding this physiological window can lead to adverse outcomes. At supraphysiological concentrations, MitoQ may paradoxically act as a pro-oxidant through redox cycling, which intensifies oxidative stress and compromises cellular viability [49]. This biphasic response underscores the importance of precise dose titration, especially in clinical settings and when applied across different species. As summarized in Table 1, these dose-dependent effects are evident across animal studies: in roosters, 150 nM MitoQ improved motility and ATP levels, whereas 200 nM led to increased ROS production [37]; in bulls, supplementation up to 20 nM provided no protective benefit and, in some cases, exacerbated oxidative stress [29,30]. By contrast, goats exhibited clear improvements in motility and membrane integrity at nanomolar doses [34,35], and boar sperm benefitted from variable doses spanning under 40 µM range [40].

In humans, experimental nM–sub-µM concentrations increased total motility but did not improve viability [39]. These findings highlight both species-variability and the narrow therapeutic window of MitoQ. Moreover, the variability in mitochondrial characteristics, such as density, membrane potential, and intrinsic antioxidant systems, demands species-specific optimization. To ensure safe and effective application, comprehensive toxicity and safety evaluations are essential. Importantly, clinical studies in humans (Table 4) confirm that oral MitoQ doses of 20–80 mg/day are safe and well tolerated across multiple populations, including patients with hepatitis C, Parkinson’s disease, and older adults, although disease-modifying effects were limited [19,50,51,52,53]. This data provides confidence in its safety profile but also emphasizes that efficacy and toxicity thresholds may differ between systemic administration and gamete-focused applications. These studies should particularly focus on long-term reproductive outcomes, which are crucial for both regulatory approval and successful clinical translation.

The implications extend further into conservation biology, where cryopreservation plays a vital role in safeguarding endangered species [34]. MitoQ’s capacity to maintain sperm integrity through repeated freeze–thaw cycles support its application in wildlife conservation and ex situ breeding programs [34,36,48]. As shown in Table 1, this protective effect has been demonstrated in multiple species: goat sperm benefitted from improved motility and mitochondrial activity at 100–150 nM [34,35], while ram semen stored at 10–100 nM maintained better motility during chilling [35]. Boar sperm also exhibited improved post-thaw viability and reduced lipid peroxidation across variable doses [40], highlighting its relevance for aquatic biodiversity preservation. In these contexts, where gamete resources are often limited and irreplaceable, MitoQ offers a means to preserve genetic diversity and support species recovery efforts [34].

### 3.1. Applications in Assisted Reproduction and Livestock Breeding

The wide-ranging protective effects of MitoQ present significant potential across various areas of reproductive and biological science. Within Assisted Reproductive Technologies (ART), MitoQ supplementation may offer transformative benefits, especially for individuals experiencing infertility associated with oxidative stress or undergoing multiple IVF cycles, where sperm quality is often compromised [8,9,10,13]. By enhancing mitochondrial function, MitoQ has the potential to improve fertilization rates, embryo development, and overall pregnancy outcomes [12,36,48]. This, in turn, may reduce the need for invasive procedures or reliance on donor sperm, offering a more natural and effective path to conception [36]. In livestock breeding, MitoQ’s ability to enhance post-thaw sperm motility and viability makes it a valuable tool for improving the success of artificial insemination. Moreover, its demonstrated efficacy across a range of species, including goat, canine, human, bulls, pigs, rooster and sheep, underscores its versatility and practical utility in animal husbandry. Consequently, by improving reproductive efficiency, MitoQ could not only help reduce breeding costs but could also accelerate genetic gain, thereby contributing to more sustainable and productive agricultural practices [34,36,39,40,48,53].

### 3.2. Applications and Future Directions

MitoQ presents a wide array of promising applications across fertility preservation, livestock breeding, bioengineering, and mechanistic research, with future directions poised to expand its utility even further [34,36,48]. In the realm of fertility preservation, MitoQ holds particular value in scenarios where sperm quality is compromised. For cancer patients undergoing chemotherapy or radiation, sperm banking prior to treatment is a common strategy. Supplementation with MitoQ may enhance post-thaw sperm quality, thereby increasing the success of fertility preservation efforts [36]. Similarly, in cases of age-related decline, where oxidative stress in sperm intensifies with advancing paternal age, MitoQ may help mitigate deterioration and improve outcomes in ART [8,9,10,13,36]. Routine incorporation of MitoQ into cryopreservation protocols could also reduce oxidative damage, enhance fertilization rates, and lower the incidence of cycle failure. In livestock and commercial breeding programs, MitoQ-enriched semen extenders offer the potential to improve post-thaw sperm function and boost conception rates [33,36,48].

## 4. Conclusions

MitoQ marks a notable breakthrough in sperm cryopreservation by delivering targeted antioxidant protection directly within the mitochondria, the primary site of ROS generation. Its distinctive chemical design enables selective accumulation in the mitochondrial matrix, allowing for the efficient neutralization of harmful ROS while preserving key functions such as membrane potential, ATP synthesis, and overall sperm viability. Across a wide range of species, including humans, livestock, and endangered animals, experimental studies consistently show that MitoQ enhances post-thaw sperm motility, viability, DNA integrity, and fertilization potential. Beyond cryopreservation, MitoQ holds promise for broader applications in assisted reproductive technologies, animal breeding programs, and conservation efforts. However, its efficacy is dose-dependent, and inappropriate concentrations may trigger pro-oxidant effects, underscoring the need for precise dosing strategies. Advances in delivery systems, such as nanoparticle encapsulation, hydrogel matrices, and synergistic combinations with other cryoprotectants, offer exciting avenues to improve the stability, bioavailability, and mitochondrial targeting of this approach. Future research should aim to unravel the molecular pathways underlying MitoQ’s mitoprotective actions, evaluate its long-term impact on reproductive outcomes, and establish standardized protocols for clinical and agricultural use. With its proven ability to counteract oxidative stress-induced sperm damage, MitoQ stands at the forefront of innovation in mitochondrial therapeutics and cryobiology, offering new possibilities for enhancing fertility preservation across species.

## Figures and Tables

**Table 1:** Experimental and clinical uses of MitoQ in sperm biology.

	Species	Application	Concentration	Outcome
Rezaei et al. (2023) [34]	Goat (Frozen semen)	Cryopreservation; MitoQ ± trehalose in extender	100–1000 nM	Improved post-thaw viability, plasma membrane integrity, mitochondrial activity; dose-dependent benefits.
Yi et al. (2024) [35]	Goat (Frozen semen)	Cryopreservation with five antioxidants including MitoQ	150 nM	Enhanced viability, membrane integrity, mitochondrial activity
Farshad et al. (2025) [36]	Canine (Frozen semen)	Cryopreservation with MitoQ + antifreeze protein III	Not specified (nM range)	Improved motility and post-thaw survival
Sun et al. (2022) [37]	Rooster (Frozen semen)	Added to cryopreservation extender	50–200 nM (optimal 150 nM)	150 nM improved motility, viability, ATP 200 nM increased ROS
Masoudi et al. (2024) [38]	Ram (Chilled semen)	Cold storage	10 nM, 100 nM	Improved motility and preserved sperm quality during chilling.
Câmara et al. (2022) [30]	Bull (Frozen semen)	Added to extender	0.2, 2, 20 nM	No improvement 20 nM increased ROS.
Al-Tarayra et al. (2024) [39]	Human (Chilled semen)	Swim-up preparation	nM to <1 µM (experimental doses)	Increased total mobility no effect on viability.
Shi et al. (2022) [40]	Boar sperm (Frozen semen)	Cryopreservation protocols	<40 μM	Improved post-thaw viability Reduced lipid peroxidation.
Elkhawagah et al. (2024) [25]	Horse sperm (Frozen semen)	Cryopreservation protocols	25, 50, and 100 nM	at 25 nM improved sperm motility, while 200 nM impaired

**Table 2:** Applications of MitoQ in other reproductive/gamete contexts.

	Application	Concentration	Outcome
Feng et al. (2024) [42]	Bos taurus oocytes (IVM from culled cows)	1–5 µM	Improved maturation and blastocyst rates; enhanced mitochondrial activity; reduced ROS
Tsui et al. (2023) [43]	Mus musculus oocytes (oxidative stress model)	µM range	Improved spindle integrity and chromosomal stability; increased survival under stress
Ferreira et al. (2025) [44]	Bos taurus oocytes (IVF media supplementation)	1 µM	Enhanced embryo development and mitochondrial function; reduced oxidative damage

**Table 3:** Applications of MitoQ in preclinical disease models.

	System	Outcome
Liu et al. (2018) [45]	Rodent ischemia–reperfusion (liver, gut, kidney)	Reduced ROS, tissue damage, apoptosis.
Graham et al. (2009) [46]	Animal cardiovascular models	Improved endothelial function and reduced oxidative stress.
Al-Tarayra et al. (2024) [39]	Human sperm in vitro	Enhanced motility and mitochondrial activity without harming viability.
Shinn und Lagalwar (2021) [47]	Neurodegenerative disease models (preclinical)	MitoQ reduced oxidative stress and improved neuronal resilience.

**Table 4:** Clinical applications of MitoQ in human studies.

	Population	Dose	Outcome
Gane et al. (2010) [50]	Chronic hepatitis C patients	40 mg/day	No significant antiviral effect; safe.
Snow et al. (2010) [51]	Parkinson’s disease trial	40–80 mg/day	No slowing of progression; well tolerated.
Rossman et al. (2018) [19]	Older adults (endothelial function)	20 mg/day for 6 weeks	Improved brachial artery flow-mediated dilation.
Braakhuis (2018) [52]	Multiple human trials	20–80 mg/day	Improved oxidative stress markers

## Data Availability

No new data were created or analyzed in this study. Data sharing is not applicable to this article.
